# When Bacteria Lose a Single DNA Base, Aphids Suffer

**DOI:** 10.1371/journal.pbio.0050126

**Published:** 2007-04-10

**Authors:** Mary Hoff

Imagine having bacteria dictate how well you fare under extreme conditions. For the aphid*Acyrthosiphon pisum*, that’s the price it pays for getting the nutrients it needs. This little insect, which survives by sucking juices out of the stems of grain crops and other vegetation, depends on a bacterial sidekick,*Buchnera aphidicola*, for amino acids it can’t get from plants. The aphid in turn provides the bacterium with energy and carbon as well as shelter inside specialized cells.

Such interdependent relationships are not unusual in the natural world. What is unusual, report Helen Dunbar, Nancy Moran, and colleagues in a new study, is that a single point mutation in*Buchnera*’s genome can have consequences for its aphid partner that are sometimes detrimental, and sometimes beneficial.

The issue in this instance is*Buchnera*’s—and so*A. pisum*’s—ability to handle heat. When exposed to high temperatures,*Buchnera*activates special “heat-shock” genes, whose products help to protect proteins from heat-related degradation. At least, that’s how it’s supposed to go. By using microarrays to assess activity of*A. pisum*and*Buchnera*genes, the researchers discovered that after a four-hour exposure to 35 °C, some of their laboratory strains of*Buchnera*upregulated the heat-shock genes, but others did not. Further analysis showed the genetic basis for the difference: a single missing nucleotide in an adenine-filled stretch of DNA, called a promoter, that’s involved in activating the heat-shock gene. Testing at a range of temperatures from 15 °C to 35 °C showed that activation of the heat-shock gene was consistently lower in the lines with the missing nucleotide than in the normal bacteria.

What does this mean for*A. pisum*’s ability to tolerate tough conditions? To answer that, the researchers asked whether exposing juvenile aphid hosts of*Buchnera*with either long or short promoters to four hours of high temperatures (35 or 38 °C) affected their ability to reproduce. They found that few of the aphids with bacteria bearing short promoters reproduced after the heat treatment, while those with bacteria bearing the longer promoters had no trouble. In addition, aphids that had been exposed to the high temperatures and had the short-promoter–bearing bacteria weighed less as adults and had far fewer*Buchnera*inside them than did their counterparts with long-promoter–bearing bacteria.

Given these seeming huge disadvantages to dropping a single adenine, it’s hard to believe the mutation could last long in a*Buchnera*population. Yet by sequencing and comparing the*Buchnera*associated with various*A. pisum*lines, the researchers discovered that the short-promoter option had arisen and been fixed twice in laboratory stock and was also found at frequencies of 21% and 13%, respectively, in bacteria in field-collected aphids from Wisconsin and New York.

**Figure:**
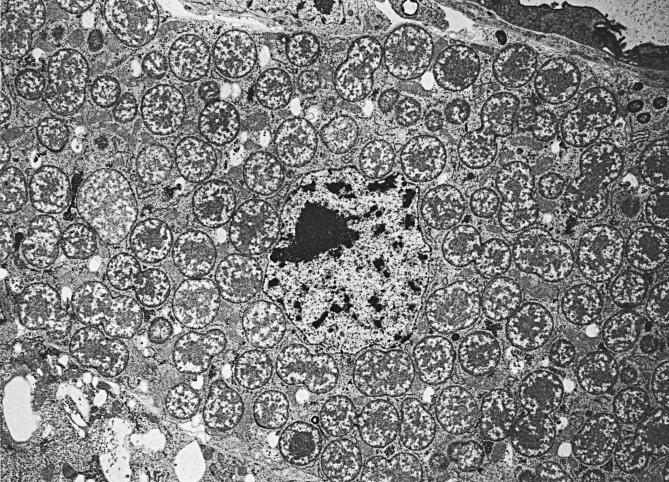
Symbionts (Buchnera aphidicola) within a bacteriocyte of a pea aphid (Acyrthosiphon pisum). The central object is the host nucleus; Buchnera cells are round and packed into the cytoplasm. (Photo: J. White and N. Moran, University of Arizona)

Population genetic theory predicts that when a mutation is maintained in a population at high frequencies, it likely confers some benefit to its bearer. What could be the advantage of carrying a gene that causes one to lose one’s ability to reproduce at high temperatures?

A clue to the answer comes from the wild populations in which the mutation was not found: those living in Arizona and Utah. Could the bacterial mutation confer a competitive advantage that’s only relevant in cooler climates? To find that out, the researchers performed a second test using a range of four-hour exposure temperatures. They discovered that short-promoter-bacteria–bearing aphids produced progeny faster than did the normal ones when raised at 15 °C or 20 °C. Thus, though aphids containing bacterial symbionts with the heat-shock–promoter mutation fare worse than normal aphids after exposure to high temperatures, they do better under cool conditions, giving the mutation a selective advantage that causes it to be maintained in the population.

In addition to their explorations of*A. pisum*and its*Buchnera*, Moran’s team also looked for and found multiple-adenine stretches related to heat-shock genes in*Buchnera*symbiotic with other aphid species. This offers fertile ground for further study of the intriguing interplay among aphids, bacteria, and temperature.

